# Evaluating the Compatibility of Three Aluminum Salt-Adjuvanted Recombinant Protein Antigens (Trivalent NRRV) Combined with a Mock Trivalent Sabin-IPV Vaccine: Analytical and Formulation Challenges

**DOI:** 10.3390/vaccines12101102

**Published:** 2024-09-26

**Authors:** Prashant Kumar, Atsushi Hamana, Christopher Bird, Brandy Dotson, Soraia Saleh-Birdjandi, David B. Volkin, Sangeeta B. Joshi

**Affiliations:** Department of Pharmaceutical Chemistry, Vaccine Analytics and Formulation Center, University of Kansas, Lawrence, KS 66047, USA

**Keywords:** pediatric combination vaccine, non-replicating rotavirus vaccine, Sabin inactivated polio vaccine, formulation, compatibility, stability, preservatives, adjuvant, ELISA

## Abstract

In this work, we describe compatibility assessments of a recombinant, trivalent non-replicating rotavirus vaccine (t-NRRV) candidate with a mock trivalent Sabin inactivated polio vaccine (t-sIPV). Both t-sIPV and t-NRRV are incompatible with thimerosal (TH), a preservative commonly used in pediatric pentavalent combination vaccines (DTwP-Hib-HepB) distributed in low- and middle-income countries (LMICs), preventing the development of a heptavalent combination. The compatibility of t-NRRV with a mock DTwP-Hib-HepB formulation is described in a companion paper. This case study highlights the analytical and formulation challenges encountered when combining a mock t-sIPV vaccine (unadjuvanted) with Alhydrogel^®^ (AH) adjuvanted t-NRRV. Selective and stability-indicating competition ELISAs were implemented to monitor antibody binding to each of the six antigens (±AH). Simple mixing caused the undesired desorption of t-NRRV from AH with the concomitant binding of t-sIPV to AH. Although the former effect was mitigated by dialyzing sIPV bulks, decreased sIPV storage stability was observed at accelerated temperatures in the bivalent combination with a rank-ordering of P[8] > P[6] > P[4] and sIPV3 > sIPV2 > sIPV1. The compatibility of AH-adsorbed t-sIPV with alternative preservatives was evaluated, and parabens (methyl, propyl) were identified for potential use in this multi-dose bivalent formulation. Along with a companion paper, the lessons learned are discussed to facilitate the future formulation development of pediatric combination vaccines with new antigens.

## 1. Introduction

Combination vaccines contain multiple antigens in a single vaccine dose, thereby providing increased disease protection with fewer injections. This simplified vaccination schedule results in numerous public health, economic, and societal benefits [1,2,3]. For example, vaccination with pentavalent pediatric combination vaccines containing inactivated whole-cell pertussis antigen (wP) (i.e., DTwP-Hib-HepB) has improved vaccine access and compliance in LMICs [1,4,5]. Attempts to formulate a hexavalent pediatric combination vaccine for LMICs, by adding inactivated polio vaccine (t-IPV antigens) to DTwP-Hib-HepB, has historically failed due to numerous technical challenges, including the incompatibility of t-IPV antigens with the preservative thimerosal (TH) used in the current manufacturing process to produce wP [6,7,8]. This has led to recent efforts to prepare inactivated wP bulks without TH, which has facilitated the successful development of this hexavalent combination [6,7,8]. 

As part of multi-dose combination vaccine formulation development with a recombinant protein-based rotavirus vaccine candidate (t-NRRV antigens; see below), we previously evaluated the compatibility of t-NRRV with DTwP-Hib-HepB antigens, as described in a companion paper [9]. The instability of t-NRRV was shown to be caused by selected components of the pentavalent vaccine (i.e., certain antigens and adjuvants as well as the preservative TH) [9]. Based on these considerations, the focus of this work was to evaluate a possible bivalent vaccine combination (t-NRRV + t-sIPV), which could potentially be administered in the same vaccination schedule in LMICs along with the currently used pentavalent (DTwP-Hib-HepB) vaccine. Since the pentavalent pediatric combination vaccine is a multi-dose formulation (to further lower costs), the bivalent vaccine formulation would also require a preservative, but an alternative preservative must be identified since both t-sIPV and t-NRRV are incompatible with TH (see Section 4).

As a first step, we prepared a mock trivalent sIPV formulation by utilizing three Sabin IPV antigens (types 1, 2, 3). There are two types of polio vaccines used worldwide for the prevention of poliomyelitis, namely inactivated polio vaccines (referred to as IPV or Salk) and oral polio vaccines (OPV or Sabin) [6]. The IPV contains formalin-inactivated Salk polioviruses (types 1, 2, 3) for parenteral administration, while OPV comprises Sabin live attenuated polioviruses (types 1, 2, 3) for oral administration. Both vaccines were developed in 1950s, and their proven efficacy has dramatically reduced global polio cases from hundreds of thousands in 1950s to below two thousand in 2020 (currently mainly occurring in Africa and Eastern Mediterranean countries) [6,10,11]. Owing to the high manufacturing costs, IPV has primarily been used in high-income countries (HICs), while the more affordable OPV is used in LMICs [12]. In a rare event, however, the OPV vaccine can revert in humans to its neurovirulent form, leading to vaccine-associated paralytic poliomyelitis (VAPP) [13,14]. VAPP can further mutate in humans to regain sustained transmissibility, referred to as circulating vaccine-derived poliovirus (cVDPV), with potential to cause larger scale polio outbreaks [13,14,15]. One promising approach to replace OPV vaccines is the introduction a lower-cost IPV vaccines that avoids the large-scale cultivation of wild-type polio strains as currently used in preparing conventional Salk IPV [6,16]. To this end, several vaccine manufacturers are engaged in developing Sabin IPV vaccines (i.e., sIPV made from inactivated OPV strains), and the first trivalent Sabin vaccine (t-sIPV) to receive WHO prequalification status was Eupolio^TM^ (LG Chem Ltd., Seoul, Republic of Korea) in 2020 [7]. 

We then evaluated the compatibility of a mock trivalent sIPV formulation with an injectable, next-generation rotavirus vaccine (RV) candidate. Despite the availability of four live attenuated orally delivered WHO-pre-qualified RV vaccines and other locally approved vaccines [17,18], there were still ~128,000 deaths (in 2016) in children below 5 years worldwide [19,20]. This is due to a combination of effects, including low RV vaccine coverage (~30%), high manufacturing costs, limited manufacturing capacity, and lower efficacy of RV vaccines in low-resource settings (~40–60% in LMICs vs. ~80–90% in HICs). One approach to address these issues is the development of injectable, next-generation RV vaccines with the potential to (1) be combined with other pediatric vaccines (e.g., IPV or pentavalent DTP-Hib-HepB) [21], (2) have lower costs and be more affordable, and (3) be safer (no vaccine-induced intussusception) with a higher efficacy in high-morbidity settings [21,22,23,24]. To this end, since RV vaccines (and other vaccines, i.e., DTaP, Hib, and HepB) can be administered with IPV at the same doctor’s visit per CDC guidance [25], this non-interference of IPV immune response with RV antigens administered at the same visit suggests the possibility of non-interference when administered in a combined vaccine [17,22]. 

To prepare an injectable, next-generation rotavirus vaccine (RV) candidate, we utilized three non-replicating RV vaccine (t-NRRV) recombinant protein antigens formulated with an aluminum hydroxide adjuvant (Alhydrogel^®^, AH). Each of the three NRRV antigens (P[4], P[6], P[8]) is produced recombinantly in *E. coli* as a fusion protein containing (1) universal tetanus toxoid CD4+ T-cell epitope P2 and (2) a truncated ΔVP8* protein (soluble truncated version of VP8* proteolytically cleaved from VP4 protein from three different RV serotypes, P[4], P[6], P[8]) joined together via a GSGSG linker, as described in detail elsewhere [26,27]. Early-phase clinical studies conducted by PATH demonstrated promising results for t-NRRV; however, interim analysis from a recent Phase 3 clinical trial in Africa demonstrated insufficient evidence to confirm superior protection against severe RV gastroenteritis compared to the currently licensed oral RV vaccines [28]. Nonetheless, in this work, t-NRRV antigens serve as a representative new recombinant protein-based antigen to evaluate the analytical and formulation challenges encountered upon combining new vaccine candidates with the existing t-IPV or t-sIPV vaccines.

Three major formulation and analytical challenges are described in this work, including (1) development and set up of selective and stability-indicating competitive ELISAs for each of the six antigens (t-NRRV, t-sIPV) in terms of binding to a specific antibody in the presence of aluminum salt adjuvants, (2) the compatibility (immediately after formulating) and stability (during longer term storage) of trivalent AH-adsorbed NRRV antigens with a mock trivalent sIPV vaccine formulation as measured by the AH adsorption and antibody binding of each of the six antigens, and (3) the screening of eight different antimicrobial preservatives (APs) for their destabilizing effects on the sIPV antigens and the down-selection of potential alternative APs to replace TH for future bivalent vaccine multi-dose formulation development work. 

## 2. Materials and Methods

### 2.1. Materials 

The three NRRV antigens (P[4], P[6], and P[8]) were provided by SK Biologics (Seongnam-si, Republic of Korea) and PATH (Seattle, WA, USA) in PBS buffer, stored frozen and thawed prior to use. The three Sabin inactivated poliovirus (sIPV types 1, 2, and 3) bulks were provided by Batavia Biosciences (Leiden, The Netherlands) in M199 medium stored at 2–8 °C. Capture and detection antibodies used in the competition enzyme-linked immunoassays (ELISAs), system suitability controls, and reference standards were purchased from Batavia Biosciences (The Netherlands) for three sIPV antigens and Precision Antibody (Columbia, MD, USA) for the three NRRV antigens. Alhydrogel^®^ (AH) adjuvant was purchased from InvivoGen (San Diego, CA, USA). All reagents and chemicals used in the study were of analytical grade or higher and purchased from Sigma-Aldrich (St. Louis, MO, USA).

### 2.2. Competitive ELISA Development for t-NRRV and t-sIPV Antigens

Competitive ELSAs for the three NRRV antigens (P[4], P[6], and P[8]) capable of measuring them both in the presence (adsorbed) and absence of AH were transferred from PATH, USA, as described in detail elsewhere [29,30]. The capture monoclonal antibodies were obtained from Precision Antibody, USA (i.e., mAbs 7H7 and 3G11 bind linear epitopes on P[8] and P[6] antigens, respectively, while mAb 13A1 binds a conformational epitope on P[4]), as described in detail elsewhere [29,30]. Each of the steps for the competitive ELISAs for each of the three NRRV antigens in a combination vaccine formulation is described in detail in our companion paper [9]. 

Competitive ELISAs for the three sIPV antigens were developed, for measuring in the presence (adsorbed) and absence of AH adjuvant, in terms of Sabin D-antigen units (SDU) using capture antibodies (specific for sIPV types, 1, 2, and 3). The competitive ELISAs for the three sIPV antigens were performed using similar steps as described for the NRRV antigens’ ELISA in our companion paper [9], but used the capture antibodies specific for each sIPV types 1, 2, and 3 purchased from Batavia Biosciences and a commercial detection antibody (Invitrogen G21234). 

The competitive ELISAs for both t-NRRV and t-sIPV antigens were utilized for measuring the total antigen (bound + unbound to AH adjuvant) in a sample, and the percentage of adsorbed antigen by measuring the supernatant (unbound) and pellet (bound) fractions after centrifugation, as described in detail in our companion paper [9]. The selectivity and stability indication of each ELISA was confirmed (using a procedure adapted from our companion paper [9]) before utilizing them for the compatibility and stability evaluations of the combined bivalent vaccines. Briefly, selectivity was tested in the absence of any stress condition, by comparing the known concentration of each (1) alum-adjuvanted specific antigen (assay standard) vs. (2) alum-adjuvanted specific antigen along with the non-specific antigens, and (3) alum-adsorbed non-specific antigens minus the specific antigen. The stability indication of each ELISA was tested by applying thermal stress to alum-adjuvanted antigens for specific time (NRRV P[4], P[6], and P[8] antigens at 50 °C for 40 min; and sIPV types 1, 2, and 3 antigens at 70 °C for 30 min), and comparing them with control samples of the same formulation stored at 2–8 °C for the same time. 

### 2.3. Compatibility Studies

The compatibility assessment of AH-adsorbed t-NRRV (P[4], P[6], and P[8]) with t-sIPV (types 1, 2, and 3; no adjuvant) was carried out by the simple mixing of 2X concentrations of both vaccines to prepare 1X bivalent vaccine (t-NRRV + t-sIPV). To prepare 2X t-NRRV, first, mock 6X t-NRRV vaccine formulations were prepared by adding calculated amounts of each NRRV antigen (P[4], P[6], and P[8]) stock solution with AH adjuvant in low-phosphate PBS (phosphate-buffered saline contained 0.5 mM phosphate, 150 mM NaCl, pH 7.0) to a target pH of 7.0 and incubated at 2–8 °C overnight. Then, the three monovalent AH-adsorbed NRRV formulations were mixed in equal volume to prepare 2X t-NRRV vaccine formulation. The preparation of 2X sIPV trivalent mock vaccine formulation was carried out by adding the calculated amounts of each sIPV bulk (types 1, 2, and 3) to 1X M199 medium (containing ~19 mM phosphate buffer) to a target pH of 7.0. 

To prepare the bivalent vaccine formulation, 3.5 mL each of the 2X t-NRRV and 2X t-sIPV formulations were then mixed to prepare 7 mL of 1X bivalent vaccine (60 µg/mL of each NRRV (P[4], P[6], and P[8]), 20 SDU/mL of sIPV1, 32 SDU/mL of sIPV2, 36 SDU/mL of sIPV3, 1.125 mg/mL AH, ~10 mM phosphate in 0.5X M199 medium at a target pH of 7.0) and stored in a 15 mL conical tube at 2–8 °C overnight (i.e., at time zero), followed by the ELISA analysis of each of the NRRV and sIPV antigens for antigen integrity (antibody binding) and degree of AH adsorption. 

The investigation of the causes of the desorption of NRRV antigens from AH was carried out by mixing 3.5 mL of 2X concentration of AH-adsorbed mock trivalent NRRV vaccine with 3.5 mL of 2X trivalent sIPV formulated using dialyzed sIPV to remove the M199 medium and replace it with low-phosphate PBS buffer to prepare 7 mL of low-phosphate PBS bivalent formulation. The formulation contained each NRRV (P[4], P[6], and P[8]) antigen at 60 µg/mL, 20 SDU/mL of sIPV1, 32 SDU/mL of sIPV2, 36 SDU/mL of sIPV3, AH at 1.125 mg/mL, ~0.5 mM phosphate PBS buffer at a target pH of 7.0 and was stored in a 15 mL conical tube at 2–8 °C overnight (i.e., at time zero), followed by the ELISA analysis of each NRRV and sIPV antigen, as described above.

### 2.4. Stability Studies

Real-time (2–8 °C) and accelerated stability studies (15, 25, 37 °C) were carried out using (1) mock bivalent vaccine formulations (after the simple mixing of AH-adsorbed t-NRRV and mock t-sIPV vaccine as described above), (2) mock low-phosphate PBS bivalent vaccine formulations (after mixing of AH-adsorbed t-NRRV and t-sIPV vaccine prepared using sIPV bulks dialyzed into low-phosphate PBS buffer as described above), and (3) control formulations of each without mixing together (i.e., t-NRRV control contained AH-adsorbed t-NRRV and t-sIPV control contained AH-adsorbed t-sIPV; both controls were prepared in 0.5 mM phosphate PBS buffer). For these stability studies, 2.5 mL of each formulation was dispensed in 5 mL sterile glass vials, stoppered with rubber stoppers, and crimped with aluminum seals and was stored upright at the above-mentioned temperatures. Stability losses by competitive ELISA and the degree of adsorption for each antigen to the aluminum salt adjuvants were measured as described above.

### 2.5. Stability Profile of AH-Adsorbed t-sIPV Antigens with Different Antimicrobial Preservatives (APs)

The stability of t-sIPV antigens with eight different individual preservatives was studied by mixing 1X concentrations each of the AH-adsorbed t-sIPV (types 1, 2, and 3) sample with a preservative stock solution to prepare 0.5X t-sIPV formulation (sIPV1 at 10, sIPV2 at 16 and sIPV3 at 18 SDU/mL, 1.125 mg/mL AH, in 10 mM phosphate containing the M199 medium at a target pH of 7.0). The target preservatives’ concentrations were as follows: 0.25 mM thimerosal (TH), 72.4 mM 2-phenoxy ethanol (2-PE), 53.2 mM phenol (PH), 26.8 mM chlorobutanol (CB), 27.8 m-cresol (MC), 92.5 mM benzyl alcohol (BA), 11 mM methyl paraben, and 0.6 mM propyl paraben (MP+PP). Most of the preservatives were targeted to be at their highest in-use concentration [31], except for methyl paraben and propyl paraben, which were used at their maximum solubility levels at the formulation pH. For these studies, 1 mL of each multi-dose formulation was filled in 2 mL glass vials, stoppered, and stored upright at 2–8 °C overnight before incubating at 2–8 °C and 25 °C for 3 months and 37 °C for 1 day. The stability profile of each AH-adsorbed t-sIPV antigen in the presence and absence of these preservatives was measured by relative antibody binding over time using competitive Sabin D-antigen ELISA assays, as described above. 

## 3. Results

### 3.1. Competitive ELISA Development for t-NRRV and t-sIPV Antigens

**Figure 1:**
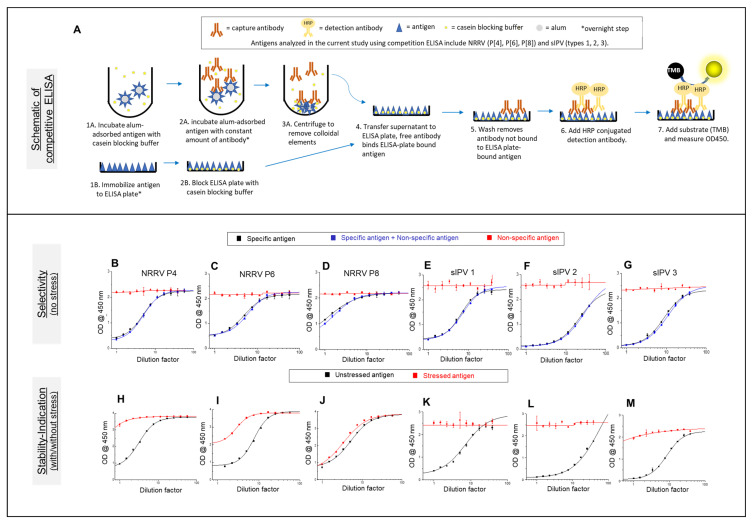
Summary of the competitive ELISA method development results for aluminum salt-adjuvanted antigens (t-NRRV and t-sIPV) for combination in a bivalent combination vaccine. Schematic of the competitive ELISA assay format (panel (**A**)); this figure was published previously in our companion paper [9] and is adapted here with permission from the *Vaccines* journal. The selectivity and stability indication results are shown for each antigen: NRRV P[4] (panels (**B**,**H**)), P[6] (panels (**C**,**I**)), P[8] (panels (**D**,**J**)), sIPV1 (panels (**E**,**K**)), sIPV2 (panels (**F**,**L**)), and sIPV3 (panels (**G**,**M**)). See the Methods Section for a description of samples and stress conditions. Data are presented as the mean ± range (n = 2). Panels (**H**–**J**) for the stability indication of NRRV antigens were previously published in our companion paper [9] and are shown here with permission from the *Vaccines* journal.

Competitive ELISAs for the three NRRV antigens (P[4], P[6], and P[8]) and the three sIPV (types 1, 2, and 3) antigens were used to assess the compatibility of each antigen in the bivalent combination formulation in the presence and absence of the AH adjuvant. A schematic of the ELISA assay format used is presented in Figure 1A. These six different competitive ELISAs measured the binding of each antigen to an antigen-specific mAb, both in AH-adsorbed and free forms, in the presence or absence of the other bivalent formulation antigens. 

For each of the three NRRV antigen (P[4], P[6], and P[8]) competitive ELISAs used in this work, concordances between NRRV in vitro ELISA readouts and in vivo neutralizing antibody response from a guinea pig model have been previously demonstrated [29,30]. These ELISA assays for each AH-adjuvanted NRRV antigen were shown to be selective (Figure 1B–D) and stability-indicating (Figure 1H–J) in the bivalent formulation. Selectivity was studied in unstressed samples by comparing known concentrations of (1) specific antigen, (2) non-specific antigens, and (3) specific antigen together with non-specific antigens. The results show the same levels of antigen binding in the case of (1) and (3), but no antigen binding was observed in case of (2) (Figure 1B–D). The stability indication of each NRRV antigen was tested by measuring the antibody binding of unstressed and heat-stressed AH-adsorbed bivalent vaccine samples (see Section 2). The results show a measurable loss in antibody binding in the case of heat-stressed vs. unstressed bivalent formulation samples for each of the three NRRV antigens (Figure 1H–J).

For each of the three sIPV antigen (type 1, 2, and 3) ELISA assays, the standard D-antigen sandwich ELISA was designed to measure unadsorbed sIPV antigens and could not be used for measuring AH-adsorbed sIPV antigens. We first attempted desorbing sIPV antigens from AH (in mock AH-adsorbed sIPV vaccine formulations) using various salts (i.e., phosphate, citrate, NaCl) combined with and without thermal treatments; however, complete antigen desorption could not be achieved. Therefore, we performed a similar competitive ELISA assay development for the sIPV antigens as described above for the t-NRRV antigens. Each of the three sIPV ELISA assays was shown to be selective (Figure 1E–G) and stability-indicating (Figure 1K–M) using the same criteria as described above for the t-NRRV antigens.

### 3.2. Compatibility of AH-Adjuvanted t-NRRV and sIPV Formulations When Mixed Together

**Figure 2:**
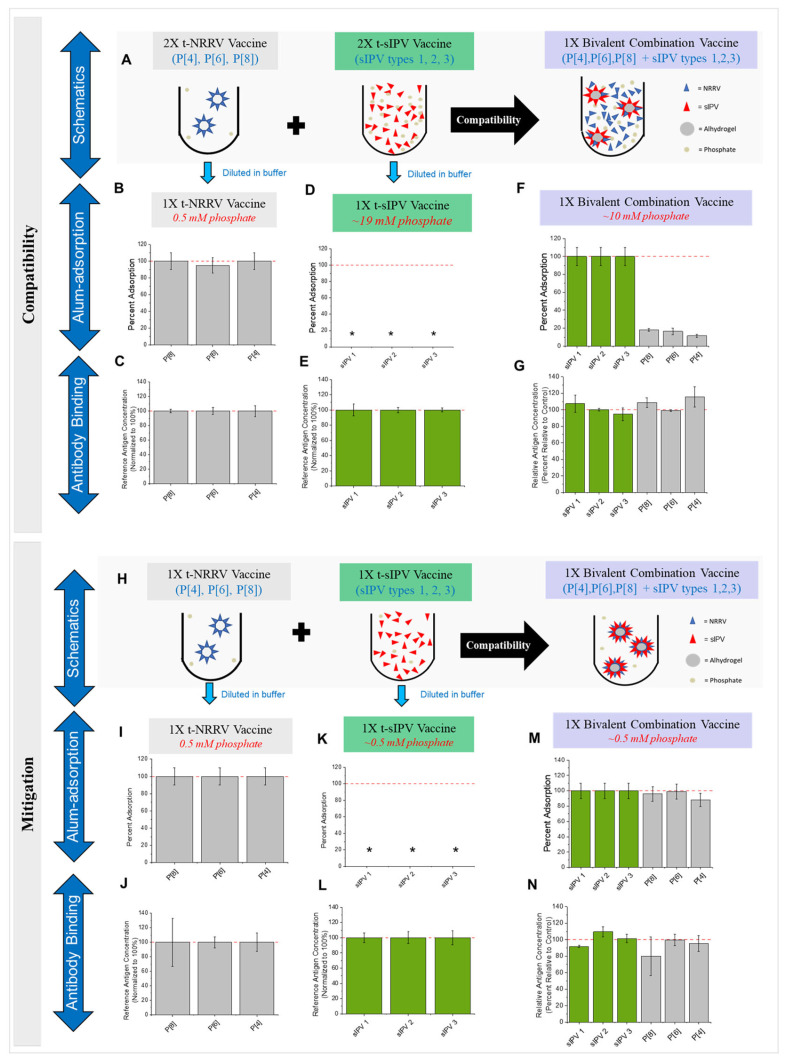
Compatibility of Alhydrogel™ (AH)-adjuvanted recombinant t-NRRV antigens (P[4], P[6], P[8]) with t-sIPV antigens (types 1, 2, and 3; no adjuvant) in a mock bivalent combination vaccine formulation (t-NRRV + t-sIPV). Panel (**A**) is schematic representation of the simple mixing together of AH-adsorbed t-NRRV antigens with t-sIPV (no adjuvant) antigens to prepare a bivalent vaccine formulation. Alum adsorption and antibody binding (relative antigen concentration normalized to 100% for each antigen) results are shown for (**B**,**C**) AH-adjuvanted t-NRRV, (**D**,**E**) a mock t-sIPV formulation, and (**F**,**G**) bivalent vaccine formulation. Panel (**H**) is schematic representation of a mitigation strategy (using sIPV bulks dialyzed in PBS to lower the phosphate buffer concentration) showing the simple mixing together of AH-adsorbed t-NRRV antigens with t-sIPV (no adjuvant) antigens to prepare a bivalent vaccine formulation. Alum adsorption and antibody binding results are shown for (**I**,**J**) AH-adjuvanted t-NRRV, (**K**,**L**) a mock t-sIPV formulation prepared using dialyzed viral bulks, and (**M**,**N**) bivalent vaccine formulation in PBS. Antibody binding and AH-adsorption for all antigens were measured by the competitive ELISA assays described in Figure 1. (*) no adsorption since AH is not present in the mock t-sIPV formulation. Red dashed line represents 100% adsorption, or 100% reference/relative antigen concentration. Data are presented as the mean ± range (n = 2) for AH adsorption and mean ± SD (n = 4) for antibody binding. When antigen adsorption to alum values was 100%, a range of ±10% was assigned based on the estimated LOQ of assay.

The simplest approach to produce this mock bivalent pediatric combination vaccine (t-NRRV + t-sIPV) is to mix together the current drug product formulations consisting of (1) three recombinant protein-based NRRV antigens adsorbed to AH adjuvant (AH-adsorbed t-NRRV in low-phosphate PBS) and (2) a solution containing the three sIPV antigens (no adjuvant in the M199-PBS solution). The target antigen and adjuvant concentrations for the t-NRRV and t-sIPV formulations were based on previous clinical experience with NRRV antigens and a literature review of WHO-pre-qualified and US FDA-approved IPV vaccines [7,8,32]. We prepared the t-NRRV and mock t-sIPV formulations at 2X target concentration (see Section 2) and mixed them together (1:1) to produce the bivalent combination vaccine formulation (see schematic in Figure 2A).

The initial compatibility results demonstrate that simple mixing is not compatible for the preparation of a bivalent vaccine. Prior to mixing, all three protein antigens in the t-NRRV control were completely adsorbed to AH (Figure 2B) and displayed targeted antibody-binding activity (Figure 2C). The three sIPV antigens in the t-sIPV control were formulated without adjuvant (in M199 media containing ~19 mM phosphate as carryover from the bulks); hence, AH adsorption was not measured (Figure 2D) and displayed targeted D-antigen antibody-binding activity (Figure 2E). Upon the mixing of these two controls to prepare the bivalent vaccine, the NRRV antigens became desorbed from AH (~80–90% desorption) (Figure 2F). Concomitantly, the three sIPV antigens initially became completely adsorbed to AH (Figure 2F). No notable differences in antigen–antibody binding (percent concentration relative to a control) for the NRRV (P[4], P[6], P[8]) and sIPV (types 1, 2, and 3) antigens were observed using in vitro ELISA after mixing (Figure 2G). Hence, incompatibility (in terms of differences in the degree of adsorption of the antigens) was observed after the simple mixing of AH-adsorbed t-NRRV and t-IPV based on the following considerations: (1) AH-adsorbed NRRV antigens are known to be more immunogenic vs. unadjuvanted antigen in guinea pigs [32] (also see Section 4) and (2) the adsorption of sIPV antigens to AH differs from the unadjuvanted form in the mock t-IPV vaccine (see Section 4).

### 3.3. Investigation and Possible Mitigation of t-NRRV Desorption from AH Adjuvant upon Mixing with sIPV Antigens

Two potential mitigation strategies were examined to retain the NRRV antigens as adsorbed to the AH adjuvant (after combining with sIPV antigens), including (1) the pre-adsorption of sIPV to AH and then buffer exchange to low-phosphate PBS and (2) the dialysis of sIPV bulks into low-phosphate PBS prior to AH adsorption. Initial studies demonstrated the latter mitigation approach was more practical to implement, and thus, it was used to prepare the bivalent combination formulation (t-NRRV + t-sIPV) (see schematic in Figure 2H). Prior to mixing, the control AH-adsorbed t-NRRV formulation showed a complete binding of the NRRV antigens to AH (Figure 2I) and targeted antibody-binding activity (Figure 2J). The control t-sIPV bulks were dialyzed into low-phosphate PBS, contained no AH adjuvant (Figure 2K), and also displayed the expected antibody-binding activity (Figure 2L). Upon mixing, essentially complete AH adsorption was observed for all three NRRV as well as for all three sIPV antigens (Figure 2M), and all antigens showed targeted antibody binding (Figure 2N), given assay variability (±20%). In summary, phosphate buffer coming from the sIPV bulks caused the desorption of the NRRV antigens from the AH adjuvant after mixing, and the pretreatment of sIPV antigens by dialysis into low-phosphate PBS (to remove M199 media and reduce the phosphate concentration to ~0.5 mM) was effective in retaining the NRRV antigens on the AH adjuvant in the bivalent mixture. The effect of this pretreatment of sIPV bulks on the stability of the six antigens in the bivalent combination was then examined, as described in the next section.

### 3.4. Storage Stability Studies of Bivalent Combination Vaccine Formulations (AH-Adjuvanted t-NRRV + t-sIPV Antigens)

We evaluated the stability profile of two different bivalent formulations under real-time (2–8 °C) and accelerated (15 °C and 25 °C) storage conditions for up to 6m, as well as under stressed (37 °C up to 2 months) conditions, as measured by competitive ELISAs for each of the six antigens. The results were compared to the control formulations of AH-adsorbed t-NRRV alone and AH-adsorbed t-sIPV alone. The two bivalent formulations included (1) the simple mixing of AH-adsorbed t-NRRV and mock t-IPV vaccine prepared from undialyzed sIPV bulks (called bivalent formulation), and (2) the mixing of AH-adsorbed t-NRRV and mock t-sIPV vaccine prepared using dialyzed sIPV bulks (to remove M199 media and reduce the phosphate buffer concentration, called low-phosphate bivalent formulation).

First, antigen adsorption to the AH adjuvant was examined for the t-NRRV and t-sIPV components of the control and bivalent formulations. The t-NRRV antigens remained ~100% AH-adsorbed in the control (AH-adsorbed t-NRRV alone) and in the low-phosphate bivalent formulation prepared using dialyzed sIPV bulks. In contrast, the t-NRRV antigens were only ~<10–20% AH-adsorbed in the bivalent formulation prepared without the dialysis of the sIPV bulks (Supplemental Figure S1). These values remained unchanged during storage at various temperatures, with one exception of an apparent increase in P[6] adsorption to AH in the bivalent formulation, a result likely due to an artifact caused by the aggregation of unbound P[6] overtime (i.e., both aggregated protein and AH adjuvant pellet during the centrifugation step of the assay). For the sIPV antigens, all three were completely bound to AH at time zero (immediately after mixing together) in the control (AH-adsorbed t-sIPV) and in the two bivalent formulations. These values did not change during storage across all temperatures with one exception of a partial loss in the binding of the sIPV3 antigen in the AH-adsorbed t-sIPV control and the bivalent formulation (prepared with undialyzed sIPV bulks) over time (Supplemental Figure S2). In summary, the preparation of the bivalent combination (t-NRRV + t-sIPV) using dialyzed sIPV bulks resulted in complete AH adsorption for all six antigens at time zero. All three of the NRRV antigens and two of three sIPV antigens remained completely adsorbed to AH during storage up to 6 months (see Section 4). 

**Figure 3:**
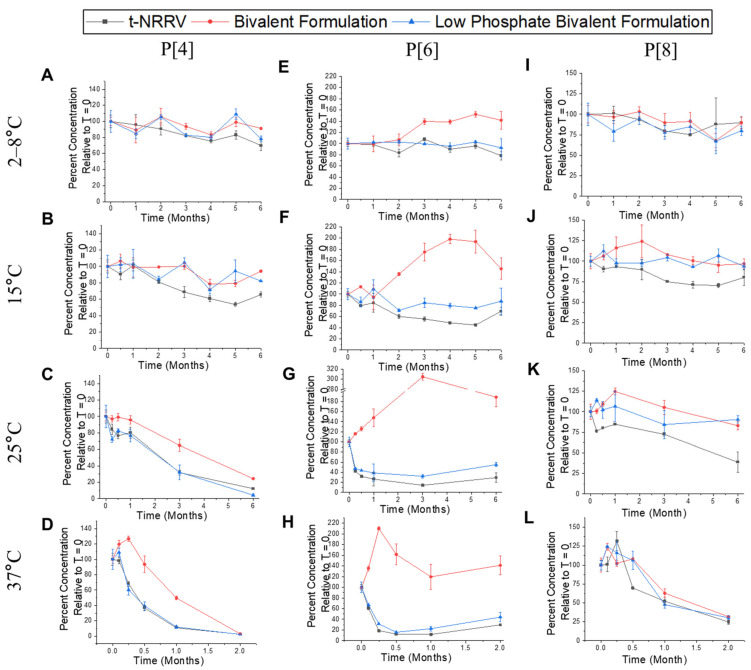
Storage stability results for each NRRV antigen (P[4], P[6], and P[8]) in an AH-adsorbed t-NRRV formulation and when added to two different bivalent combination formulations with sIPV antigens as measured by competitive ELISAs. Percent antigen binding to antigen-specific mAb (relative to T = 0) in three formulations are shown for P[4] at 2–8 °C (**A**), 15 °C (**B**), 25 °C (**C**), and 37 °C (**D**); for P[6] at 2–8 °C (**E**), 15 °C (**F**), 25 °C (**G**), and 37 °C (**H**); and for P[8] at 2–8 °C (**I**), 15 °C (**J**), 25 °C (**K**), and 37 °C (**L**). Three formulations include t-NRRV (control of AH-adsorbed t-NRRV alone), bivalent formulation (AH-adjuvant t-NRRV mixed with t-sIPV bulks at higher phosphate concentrations), and low-phosphate bivalent formulation (AH-adjuvant with t-NRRV mixed with dialyzed t-sIPV bulk with a lower phosphate concentration). Data are presented as the mean ± SD (n = 4).

Next, the antibody binding profiles for the t-NRRV and t-sIPV components of the AH-adjuvanted controls and two bivalent formulations were measured by competitive ELISAs. For the three NRRV antigens, different stability profiles were observed. For the P[4] antigen, an overall good stability was observed in all the formulations at 2–8 °C storage up to 6 months (Figure 3A). At elevated temperatures (15 °C, 25 °C and 37 °C), P[4] in the t-NRRV control and low-phosphate bivalent formulation (with dialyzed sIPV bulks) displayed similar instability profiles over time, but the bivalent formulation (undialyzed sIPV bulks) showed a trend toward relatively improved stability (Figure 3B–D). This latter result may be due to the presence of unadsorbed P[4] antigen, which is known to be more stable than the AH-adsorbed antigen [33]. For the P[6] antigen, no notable stability losses at 2–8 °C was observed in all formulations (Figure 3E). Increasing thermal stress led to similar P[6] instability trends in the t-NRRV control and low-phosphate bivalent formulation, while P[6] in the bivalent formulation displayed an unexpected increase in antibody binding at elevated temperatures (Figure 3F–H). This result is likely due to a combined effect of the presence of mostly desorbed antigen (~80% desorbed) and enhanced antibody binding to more accessible linear epitopes on the P[6] surface due to temperature-induced structural alterations. Finally, for the P[8] antigen, a good stability was demonstrated at 2–8 °C in all three formulations (Figure 3I). Increasing thermal stress showed temperature dependent instability trends in all formulations over time (Figure 3J–L). In summary, we observed (1) a good stability profiles for the three NRRV antigens in the control and two bivalent formulations at 2–8 °C with some differing degradation trends seen at elevated temperatures, (2) the presence of sIPV antigens showed no effect on t-NRRV stability, and (3) at elevated temperatures across the control and two bivalent formulations, P[8] was the most stable, P[6] was the least stable, and P[4] displayed intermediate stability.

**Figure 4:**
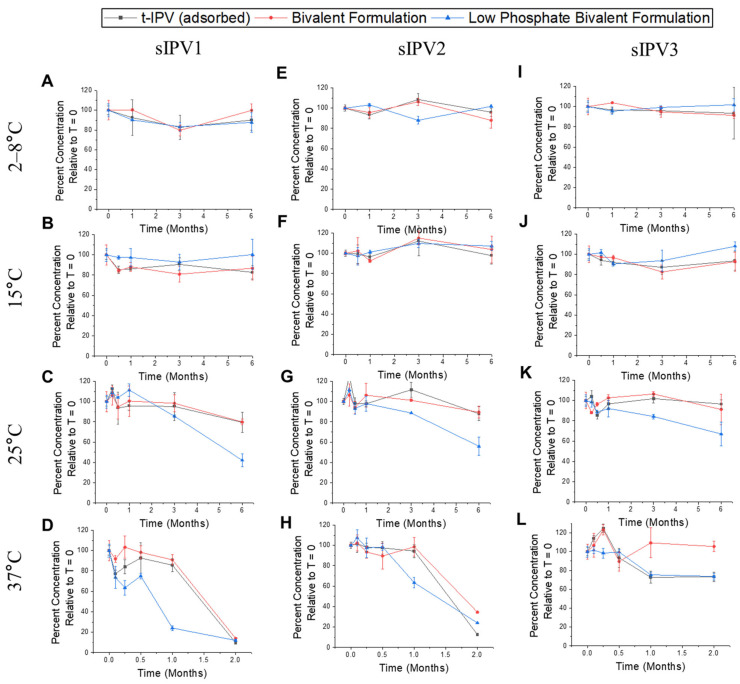
Storage stability results for each sIPV antigen (types sIPV1, sIPV2, and sIPV3) in the t-sIPV formulation (AH-adsorbed) and when added to two different bivalent combination formulations with AH-adsorbed t-NRRV antigens as measured by Sabin D-antigen competitive ELISAs. Relative antigen binding to antigen-specific mAb (relative to T = 0) in three formulations are shown for sIPV1 at 2–8 °C (**A**), 15 °C (**B**), 25 °C (**C**), and 37 °C (**D**); for sIPV2 at 2–8 °C (**E**), 15 °C (**F**), 25 °C (**G**), and 37 °C (**H**); and for sIPV3 at 2–8 °C (**I**), 15 °C (**J**), 25 °C (**K**), and 37 °C (**L**). Three formulations include t-sIPV-adsorbed (control of AH-adsorbed t-sIPV alone), bivalent formulation (AH-adjuvant t-NRRV mixed with t-sIPV bulks at higher phosphate concentrations), and low-phosphate bivalent formulation (AH-adjuvant with t-NRRV mixed with dialyzed t-sIPV bulk with a lower phosphate concentration). Data are presented as the mean ± SD (n = 4).

Finally, the stability of the three sIPV antigens were also measured in the control (AH-adsorbed t-sIPV) and two bivalent formulations. No notable losses were observed over 6 months in the three different formulations at 2–8 °C and 15 °C for sIPV1 (Figure 4A,B), sIPV2 (Figure 4E,F), and sIPV3 (Figure 4I,J) antigens. At 25 °C, no notable losses in antibody binding were observed for all three antigens in the control and the low-phosphate bivalent formulations over time; however, the low-phosphate bivalent formulation showed notable losses for all three sIPV antigens (Figure 4C,G,K). This result is presumably due to the removal of M199 and low concentrations of phosphate buffer during the dialysis of the bulks. Similar stability trends for all three sIPV antigens were observed at 37 °C (Figure 4D,H,L). In summary, we observed (1) good stability profiles for the three sIPV antigens in the control and two bivalent formulations at 2–8 °C and 15 °C; (2) at elevated temperatures, the dialysis of the sIPV bulks decreased the stability of the formulated sIPV antigens, and thus, the presence of t-NRRV antigens showed a destabilizing effect under conditions that allowed t-NRRV to remain AH-adsorbed; and (3) at elevated temperatures across the control and two bivalent formulations, sIPV1 was the least stable, sIPV3 was the most stable, and sIPV2 demonstrated intermediate stability.

### 3.5. Effect of Preservatives on Stability of Three sIPV Antigens 

**Figure 5:**
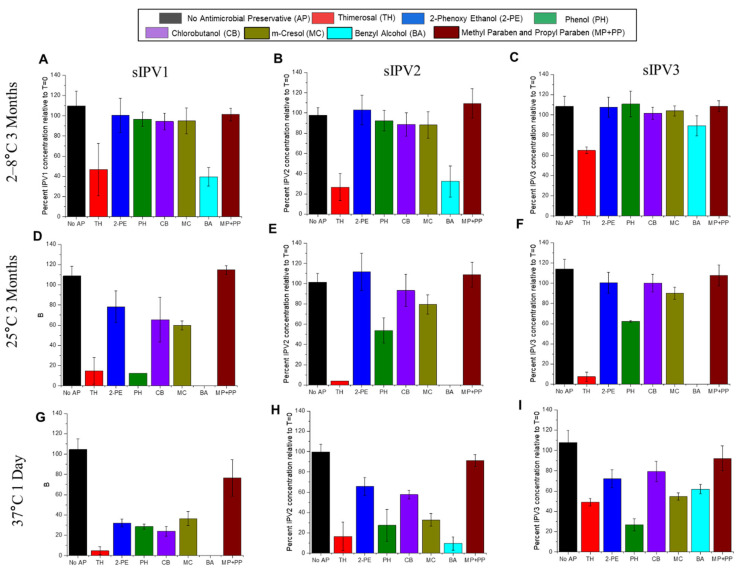
Stability profiles of AH-adsorbed t-sIPV antigens (types 1, 2, and 3) in the presence and absence of different antimicrobial preservatives (APs) as measured by Sabin D-antigen competitive ELISAs. The relative antigen–antibody binding of each AH-adsorbed sIPV antigen to an antigen-specific antibody is shown as a percentage concentration relative to time zero values after storage at 2–8 °C and 15 °C for 3 months, and 37 °C for 1 day, for sIPV1 (Panels (**A**,**D**,**G**)), sIPV2 (Panels (**B**,**E**,**H**)), and sIPV3 (Panels (**C**,**F**,**I**)) in the presence of the indicated AP. TH—thimerosal, 2-PE—2-phenoxy ethanol, PH—phenol, CB—chlorobutanol, MC—m-cresol, BA—benzyl alcohol, MP—methyl paraben, and PP—propyl paraben. Data are presented as the mean ± SD (n = 4).

Inactivated polio vaccines (IPV) are known to be unstable in the presence of thimerosal, a commonly used preservative in vaccines [6], which has led to technical challenges in the addition of IPV into the pediatric combination vaccines used in LMICs (see Section 1). t-NRRV antigens are also destabilized by thimerosal and we examined the potential use of alternative preservatives in a combination vaccine in our companion paper [9]. In this work, we evaluated the stability of three AH-adsorbed sIPV antigens with the same set of alternative preservatives for possible use in a bivalent combination vaccine (t-NRRV + t-sIPV). The relative D-antigen-binding activity of each antigen was measured using a Sabin D-antigen Competitive ELISA (see Section 2), and the stability profile for each antigen was plotted as percent relative to time zero results after storage at 2–8 °C for 3 months (Figure 5A–C), 15 °C for 3 months (Figure 5D–F), and 37 °C for 1 day (Figure 5G–I).

Compared to the no preservative control, the addition of antimicrobial preservatives caused the destabilization of AH-adsorbed antigens to varying extents depending on the sIPV type and storage temperature over 3 months. At 2–8 °C, studied over 3 months, most preservatives caused no destabilizing effect on the three sIPV antigens, including 2-phenoxy ethanol (2-PE), phenol (PH), chlorobutanol (CB), m-cresol (MC), and parabens (MP + PP). However, each of the three antigens lost D-antigen-binding activity in the presence of TH and benzyl alcohol (BA). At 25 °C, studied for 3 months, phenol (PH) also destabilized all three sIPV antigens. Finally, at 37 °C for one day, parabens (MP + PP) showed the least destabilization of the AH-adsorbed sIPV antigens. We classified the preservatives into three categories based on their destabilizing effects on the AH-adsorbed sIPV antigens across all the temperatures: (1) strongly destabilizing (thimerosal and benzyl alcohol), moderately destabilizing (phenol, chlorobutanol, and m-cresol), and weakly destabilizing (methyl paraben and propyl paraben combination, and 2-phenoxy ethanol). The three AH-adsorbed sIPV antigens displayed different relative stabilities in the presence of preservatives with sIPV3 > sIPV2 > sIPV1 in order of decreasing stability. This rank ordering matched with the stability profiles of the three AH-adsorbed sIPV antigens in the absence of preservatives (see the previous section). 

## 4. Discussion

There is growing “real-world” interest in adding new recombinant antigens to the existing pediatric combination vaccines to improve vaccine coverage and compliance, lower costs, and streamline vaccination schedules. Currently, there are no available bivalent vaccines containing both RV (currently administered orally) and IPV/sIPV (administered parenterally) antigens in the same formulation due to different administration routes. Developing new injectable, next-generation RV (iNGRV) vaccine candidates comes with several attractive value propositions, including lower manufacturing costs, more consistent vaccine supply, improved safety from intussusception, and possibly higher efficacy in LMICs [3]. Moreover, there is also the possibility to combine iNGRV vaccines with existing parenterally administered pediatric vaccines, e.g., t-sIPV and/or pentavalent (DTwP-Hib-HepB) vaccines to produce either bivalent (iNGRV + sIPV) or hexavalent (iNGRV + pentavalent) vaccines, or potentially altogether to make a heptavalent (iNGRV + sIPV + pentavalent) combination vaccine [9,21,22,34]. These presentations would also be developed as multi-dose formulations to further lower costs for vaccines targeted for use in LMICs. 

The current case study evaluated combining AH-adsorbed t-NRRV (an iNGRV candidate previously in late-stage clinical studies; see Section 1) with a mock t-sIPV vaccine to produce a possible bivalent vaccine (t-NRRV + t-sIPV). The compatibility (immediately after formulating) and storage stability (overtime at different temperatures) of each of the six antigens in the resulting bivalent vaccine was measured in terms of the AH adsorption and structural integrity of each antigen using competitive ELISA assays. The simple mixing of an AH-adsorbed t-NRRV with t-sIPV (no adjuvant) in the bivalent formulation resulted in changes in aluminum salt adjuvant binding and/or stability profiles compared to the individual control vaccines (AH-adsorbed t-NRRV, and t-IPV no AH). We outline in the following two subsections key analytical and formulation issues, including important observations and recommendations, as well as suggested future work for further developing this bivalent combination vaccine candidate.

### 4.1. Analytical Challenges and Future Work

One of the key challenges for developing a bivalent vaccine containing t-NRRV and t-sIPV antigens is developing stability-indicating assays to monitor the antigen-adjuvant interactions and storage stability profiles. Due to the complexity of combination vaccine formulations (the presence of different antigens, adjuvants, preservatives, and excipients), vaccine potency, in some cases, is still monitored by animal potency tests. As described in more detail in our companion paper [9], the majority of time required for the production of combination vaccine can be attributed to QC assays, which are time- and resource-intensive, costly, and generally show high variability. Therefore, there is growing interest to replace, reduce, and refine (referred to as the 3R principles) the use of animals in QC assays [35]. ELISAs have proven to be useful for replacing animal-based assays for QC testing of a few inactivated and recombinant vaccine antigens (e.g., IPV and Hep B, respectively) and has the potential to be a viable option to replace animal-based assays for other antigens [35,36,37,38]. For eventual use as a QC assay, antigen-specific ELISAs should not only be stability-indicating, but also correlate with immunogenicity responses in the animals of each antigen in the combination vaccine (see below).

In the current study, we utilized six different in vitro competitive ELISA assays for the three sIPV and three NRRV antigens. A sandwich D-antigen ELISA is a well-established quality control (QC) assay used for the lot release of IPV and sIPV antigens [39,40,41,42]. We developed a competitive ELISA format in this work for measuring the D-antigen content of the three sIPV antigens both in their bound and unbound forms. The three competitive ELISA assays for bulk and AH-adsorbed t-NRRV antigens have been previously described, and showed concordance with outcomes of in vivo immunogenicity studies performed in guinea pigs [29,30]. Measuring the antibody binding of antigens in AH-adsorbed form is preferred as it does not require an often-inefficient antigen desorption step (as required in the case of sandwich ELISA assays), especially during stability studies where antigen–adjuvant interactions can increase over time. Furthermore, desorbed antigens may have different conformational properties compared to the immunologically relevant AH-adsorbed antigen [29].

In this work, animal immunogenicity testing was not conducted based on the previously established links of animal immunogenicity results with the extent of the binding of these t-NRRV and sIPV antibodies. Nonetheless, in some cases, ELISA assays have been shown to be more sensitive to subtle changes in antigen structure vs. in vivo animal immunogenicity assays [6]. Hence, the outcomes for antigen stability (integrity) from the ELISA assays might not be a true representative of immunogenicity profiles of the antigens when measured using animal studies. Based on the above considerations, we recommend to utilize animal-based assays for the better understanding of the immunogenicity profiles as part of future formulation development studies. In fact, a two-step formulation development approach, i.e., using high-throughput in vitro assays (i.e., ELISA) for the initial testing of large numbers of samples, followed by the animal immunogenicity assessment of the down-selected candidate formulations, is useful, given the abovementioned practical limitations of animal assays [9].

In addition, as part of future work, physicochemical assays that correlate well with these in vitro and in vivo potency assays should be developed to better understand the multi-faceted picture of antigen conformation and stability in these formulations during storage [35]. For example, implementing such assays would help to better assess vaccine quality and the structural attributes of each antigen overtime in the presence of alum adjuvants, vaccine antigens, and other formulation components [6,29,43]. Many physicochemical assays have been established for measuring individual NRRV and sIPV antigens in monovalent formulations [6,29,43,44]; however, it will be challenging to distinguish antigen-specific signals in the presence of multiple antigens and formulation components in a bivalent vaccine.

### 4.2. Formulation Challenges and Future Work

A key challenge for developing a t-NRRV- and t-sIPV-containing bivalent formulation is the inherent sensitivity of these antigens to components of the bivalent vaccine. As described in the results, the simple mixing of the two vaccines led to the undesired desorption of t-NRRV from AH, i.e., the retention of t-NRRV antigen adsorption to aluminum salt adjuvants is anticipated to be important for immunogenicity [32]. The t-NRRV antigen desorption from the AH adjuvant after mixing with the sIPV antigens can be accounted for by the higher phosphate levels (~10 mM) in the resulting bivalent formulation, as phosphate is known to alter the net surface charge of AH from positive to negative due to the replacement of surface hydroxyl groups with phosphate ions [45]. By dialyzing the sIPV bulks to lower phosphate/M199 levels, a mitigation of t-NRRV desorption from AH was observed. 

In contrast, t-sIPV antigens were initially in solution in the monovalent vaccine without adjuvant. Upon simple mixing with AH-adjuvanted NRRV antigens, sIPV antigens were completely adsorbed to AH. This occurred with sIPV bulks when used directly (containing M199 medium and higher phosphate buffer levels) and with dialyzed sIPV bulks (into low-phosphate PBS buffer). The adsorption of sIPV antigens to AH is likely not an issue, as there are examples of alum-adsorbed IPV in standalone and combination vaccines [7,8,46]. At the same time, however, the removal of the M199 medium and lowering of the phosphate buffer levels (by the dialysis of the sIPV bulks prior to formulation into low-phosphate PBS buffer) led to the destabilization of the AH-adsorbed sIPV antigens during storage in the bivalent formulation at elevated temperatures. To this end, recommendations for future formulation development work include (1) titrating the relative stabilizing effect of the M199 medium and phosphate buffer concentration on the three t-sIPV antigens versus their relative alum desorption effect on the NRRV antigens and (2) assessing the feasibility of the sIPV antigen manufacturing process to prepare “low-phosphate” bulks.

Another formulation topic for developing a multi-dose bivalent formulation for t-NRRV + t-sIPV antigens is the identification of an antimicrobial preservative for a multi-dose presentation. Both sIPV and NRRV antigens are sensitive to TH (see Section 1), and NRRV antigens are also sensitive to the other commonly used vaccine preservative, 2-PE [6,10]. Since IPV multi-dose formulations contain 2-PE [6], but NRRV is sensitive to 2-PE, we performed an initial screening of various alternative preservatives for their effect on the stability profiles of sIPV antigens (this work) and NRRV antigens. These alternative preservatives have been previously described for potential use in multi-dose formulations of vaccine candidates since they are found in commercial parenteral products of either small molecules, protein therapeutics, or vaccines (see companion paper [9,47]). For the three sIPV antigens, we observed in this work that thimerosal and benzyl alcohol were the most destabilizing, and the combination of and methyl and propyl parabens and 2-PE were the least destabilizing. For the t-NRRV antigens, thimerosal (along with 2-PE and m-cresol) was seen to be the most destabilizing and methyl and propyl parabens to be the least destabilizing [9]. Taken together, the least destabilizing antimicrobial preservatives a multi-dose formulation of this bivalent combination vaccine would be methyl and propyl parabens. Future work requires optimizing preservative concentrations for ensuring both antigen stability and antimicrobial effectiveness. Furthermore, the cytotoxicity assessments of optimized antigen–preservative combinations should be performed along with in vivo immunogenicity studies. Finally, excipient screening could be performed to identify additives to improve t-sIPV and t-NRRV storage stability (especially in the presence of preservatives), including both in vitro potency and alum adjuvant adsorption. The ability different excipients to improve IPV/sIPV antigen stability has been recently reviewed [6].

## 5. Conclusions

In this study, we present a case study of the formulation development of a bivalent combination vaccine candidate containing three sIPV (Types 1, 2, 3) antigens (formulated in a solution without adjuvant) combined with a new recombinant protein vaccine candidate, t-NRRV, a subunit rotavirus vaccine candidate consisting of P[4], P[6], and P[8] antigens (formulated adsorbed to AH aluminum salt adjuvant). This work is a follow-up study to our companion paper [9] examining the formulation development of a hexavalent combination containing a mock formulation of a pediatric pentavalent combination vaccine used in LMICs (DTwP-HepB-Hib) with the same new recombinant protein antigens (AH-adsorbed t-NRRV). The previous study identified several incompatibility issues including the destabilization of the t-NRRV antigens due to specific components (e.g., wP antigen and TH) and antigen desorption (e.g., presence of aluminum phosphate adjuvant). The bivalent formulation examined in this work (t-sIPV + t-NRRV) was initially considered a “back-up plan” to the preferred hexavalent vaccine (see Section 1), since it was anticipated that t-NRRV + t-sIPV incompatibility would not be observed. 

Despite the simplicity of mixing together two stable formulations (t-sIPV in solution and AH-adsorbed t-NRRV), we observed incompatibilities for both the sIPV and t-NRRV antigens. We demonstrated the phosphate buffer component of the sIPV bulks in the M199 medium led to the desorption of the t-NRRV antigens from the AH adjuvant. At the same time, sIPV antigens previously in a solution all became bound to the AH adjuvant. While the removal of the M199 medium and lowering the phosphate buffer level of the sIPV bulks helped to retain t-NRRV adsorption to AH, it led to the destabilization of the sIPV antigens during accelerated storage. These results were determined by developing six selective and stability-indicating competitive ELISAs to assess the compatibility and stability of the antigens. An additional challenge included the development of a multi-dose presentation to further lower the costs of a combination vaccine, especially those targeted for use in LMICs. To this end, we also evaluated a series of alternative preservatives to replace TH as part of future multi-dose formulation work with an emphasis of t-NRRV in the companion paper [9] and sIPV antigens in this work. Together, parabens (methyl and propyl paraben) were identified as a potential option for future multi-dose formulation development efforts of this bivalent vaccine combination. 

Based on the combined results from this work and the companion paper [9], we presented two case studies outlining analytical and formulation development challenges encountered when adding a new vaccine candidate to an established pediatric combination vaccine. These two case studies should be considered “proof-of-concept” studies for developing multi-dose combination vaccine formulations since in vivo performance and long-term storage stability profiles were not evaluated. Our goal is to enable future formulation development work of new recombinant antigens that are both low-cost and have the potential for enhanced global availability and coverage by adding them to existing multi-dose formulations of pediatric combination vaccines used in LMICs. 

## Data Availability

The datasets presented in the current study are available in the KU ScholarWorks repository at https://doi.org/10.17161/1808.35555. The data are also available from the corresponding authors.

## Supplementary Materials

The following supporting information can be downloaded at: https://www.mdpi.com/article/10.3390/vaccines12101102/s1, Figure S1: AH adjuvant adsorption results for each NRRV antigen (P[4], P[6] and P[8]) in an AH-adsorbed t-NRRV formulation and when added to two different bivalent combination formulations with sIPV antigens as measured by competitive ELISAs; Figure S2: AH adjuvant adsorption results for each sIPV antigen (sIPV1, sIPV2, sIPV3) in a t-sIPV formulation (AH-adsorbed) and when added to two different bivalent combination formulations with AH-adsorbed t-NRRV antigens as measured by Sabin D-antigen competitive ELISAs.
