# Supplementary material for: Evaluating the Compatibility of Three Aluminum Salt-Adjuvanted Recombinant Protein Antigens (Trivalent NRRV) Combined with a Mock Trivalent Sabin-IPV Vaccine: Analytical and Formulation Challenges
Source: Vaccines (Basel). 2024 Sep 26;12(10):1102. doi: 10.3390/vaccines12101102 (PMC11511553; doi:10.3390/vaccines12101102)
Supplement: Supplementary file 1 [file vaccines-12-01102-s001.zip › vaccines-3185869-supplementary.pdf]

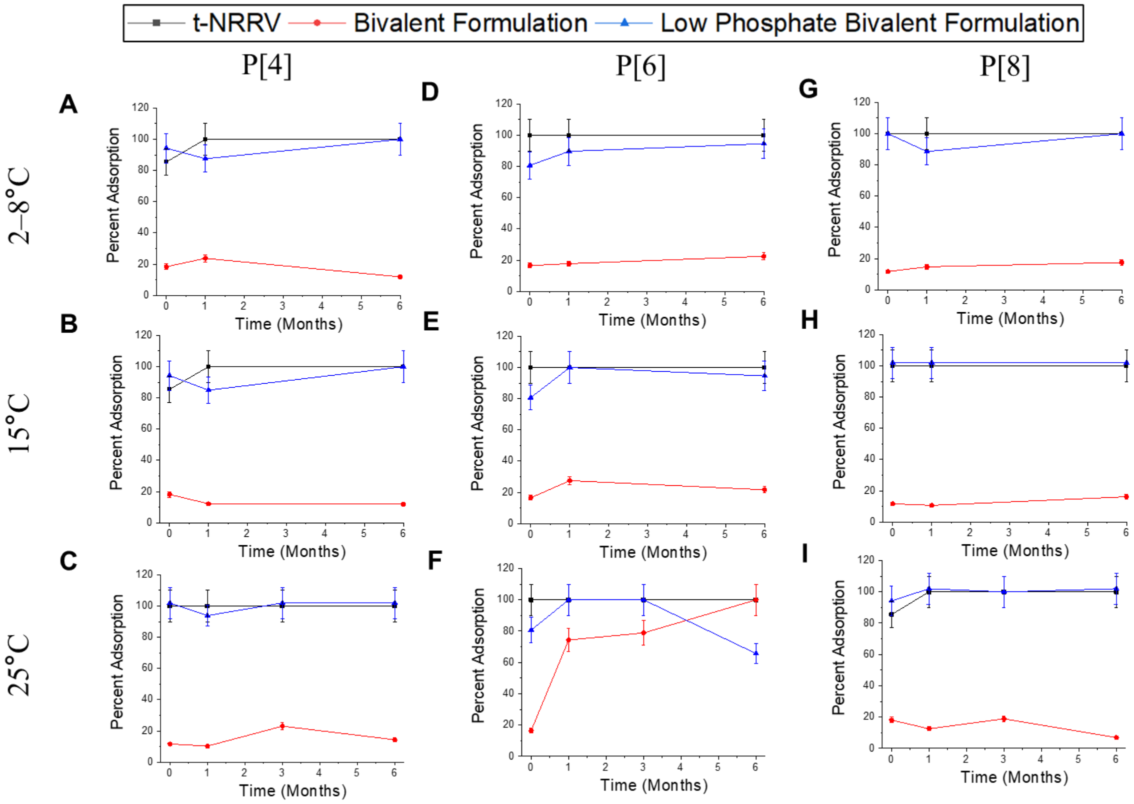

Supplemental Figure S1: AH adjuvant adsorption results for each NRRV antigen (P[4], P[6] and P[8]) in an AH-adsorbed t-NRRV formulation and when added to two different bivalent combination formulations with sIPV antigens as measured by competitive ELISAs. Percent AH-antigen binding in three formulations are shown for P[4] at 2-8°C (A), 15°C (B), 25°C (C); for P[6] at 2-8°C (D), 15°C (E), 25°C (F); and for P[8] at 2-8°C (G), 15°C (H), 25°C (I). Three formulations include t-NRRV (control of AH-adsorbed t-NRRV alone), bivalent formulation (AH-adjuvant t-NRRV mixed with t-sIPV bulks at higher phosphate concentrations) and low phosphate bivalent formulation (AH-adjuvant with t-NRRV mixed with dialyzed t-sIPV bulk with lower phosphate concentration). Data are presented as the mean ± range (n= 2).

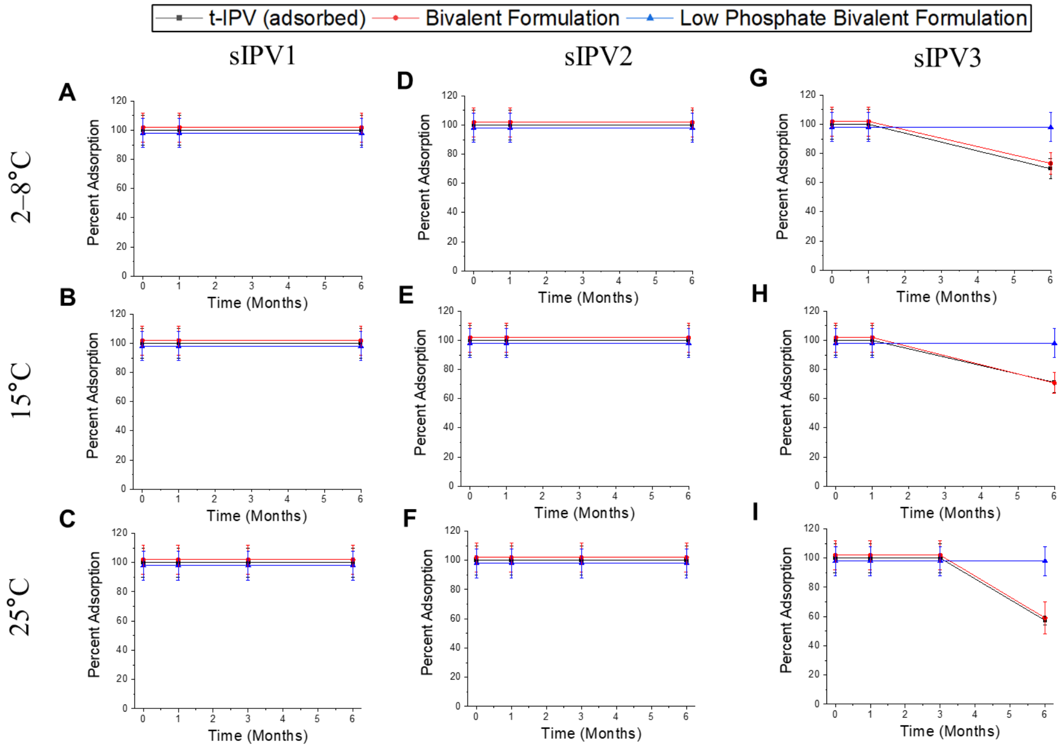

Supplemental Figure S2: AH adjuvant adsorption results for each sIPV antigen (sIPV1, sIPV2, sIPV3) in a t-sIPV formulation (AH-adsorbed) and when added to two different bivalent combination formulations with AH-adsorbed t-NRRV antigens as measured by Sabin D-antigen competitive ELISAs. Percent AH-antigen binding in three formulations are shown for sIPV1 at 2-8°C (A), 15°C (B), 25°C (C); for sIPV2 at 2-8°C (D), 15°C (E), 25°C (F); and for sIPV3 at 2-8°C (G), 15°C (H), 25°C (I). Three formulations include t-sIPV adsorbed (control of AH-adsorbed t-sIPV), bivalent formulation (AH-adjuvant t-NRRV mixed with t-sIPV bulks at higher phosphate concentrations) and low phosphate bivalent formulation (AH-adjuvant with t-NRRV mixed with dialyzed t-sIPV bulk with lower phosphate concentration). Data are presented as the mean ± range (n= 2).
